# Assessment of Patient Satisfaction of the Quality of Health Care Provided by Outpatient Services of an Oncology Hospital

**DOI:** 10.5539/gjhs.v6n5p196

**Published:** 2014-06-11

**Authors:** Anastasia Pini, Pavlos Sarafis, Maria Malliarou, Andreas Tsounis, Michael Igoumenidis, Panagiotis Bamidis, Dimitris Niakas

**Affiliations:** 1 Human Resources Department, Ministry of Health and Social Solidarity and Hellenic Open University, Greece; 2 Technological Institution of Sterea Ellada & Tutor Hellenic Open University, Greece; 3 Scientific Collaborator Technological Institution of Thessalia, Greece; 4 Centers for the Prevention of Addictions and Promoting Psychosocial Health of Municipality of Thessaloniki and Hellenic Open Uninersity, Greece; 5 Scientific Collaborator Technological Institution of Sterea Ellada, Greece; 6 Medical School AUTh & Tutor Hellenic Open University, Greece; 7 Hellenic Open University, Greece

**Keywords:** Anti-Cancer Hospital, health services, quality, patient satisfaction, oncology patients, outpatient department

## Abstract

**Aim:**

The purpose of this research is to investigate the patient’s satisfaction (patient’s satisfaction) with medical services provided in Outpatients’ Departments of a Greek Anti-Cancer Hospital in morning and afternoon clinics. The assessment of patients and identification of factors that contribute to their satisfaction will highlight the organizational and operational problems of outpatient department and assist in finding solutions to upgrade the quality of services provided.

**Material and Methodology:**

For the collection of data, a questionnaire with closed answers in a five-point scale ‘Likert’ scale was used. The questions were related to demographics, social data users, and the overall service process in the outpatient Hospital. The sample consisted of 100 patients (RR: 76%) who visited the outpatient clinic in the morning or afternoon over a month long period of time.

**Results:**

The results of our research showed that cancer patients reported a high satisfaction rate with the health services provided in outpatient department of Anti-Cancer Hospital. The highest reported levels of satisfaction were related to working with medical staff because of the special relationship of trust that patients develop with their physician. Some problems were noted during the morning shift by patients. Patients reported frustration over long waiting times to get an appointment, last minute appointments, lack of flexibility when making appointments and long waiting times before being examined by a doctor. No statistically significant relationship was found between overall satisfaction with demographics’ and other factors, although the grading services seem to be affected by the health status of patients, education and sex.

**Conclusion:**

The problems that were identified leading to less patient satisfaction were the long waiting periods to get an appointment, last minute appointments, non-flexibility in making appointments and long waiting times to be examined by the doctor. Administration should increase its efforts to upgrade the quality of health care provided to oncology patients by taking all the necessary measures.

## 1. Introduction

The expectations and demands of the citizens during crises periods from the health system are increased (Karanikolos et al., 2013). The need for quality not only in the medical-clinical work of oncology patients but all the departments providing administrative or financial services is high and there are many factors that affect patients’. One of the most modern ways of investigating the quality of health care is the measurement of the patients’ opinion as users of services. The assessment of patient satisfaction as ‘evaluation index’ is important because it helps in understanding their expectations as ‘client’ and to identify the needs and expectations of the health system. Also, a comparison of expectations with the perceived performance of services helps to identify the critical dimensions that need improvement and to find the necessary solutions. In recent years, the interest of specialists in the health sector is increasingly turning to projects and studies relating to the satisfaction of users of the care provided. It is characteristic that in the literature appear more than 1000 manuscripts each year related to patient satisfaction (Toundas et al., 2003).

In Greece, there are few of these studies, and they mainly focus on the health services provided by general hospitals. Studies of patient satisfaction in the specialized hospitals, such as cancer treatment facilities, are minimal, although a large proportion of the population is affected by cancer today. Oncology patients are a special group of patients that require specialized treatment including psychological support, and they have increased demands and expectations of the health care system. This requires the development of research that focuses on meeting their needs and measuring their satisfaction. Patient satisfaction has been the subject of many investigations in the field of health, and there are many different ways to define its meaning. Prerequisite research for the assessment includes collecting the necessary information, and ensuring it complies to sound conclusions about the quality of the service. The successful design and research results in selection of appropriate assessment indicators, specifically for health services are related to structure, process and outcome (Polyzos et al., 2005). It should be noted that in recent years the assessment of patient satisfaction has gained increasing importance as to clearly identify the overall level of performance of a health unit and defines the possible superiority compared to other equivalent (Tso & Chan, 2006; Hendriks et al., 2001; Sitzia & Wood, 1997). As an indicator for assessing health services quality, assessment of user satisfaction is a very useful tool for administrations of clinics as it provides useful information for staff when solving organizational and operational problems (Aletras et al., 2007; Saila et al., 2008; Kleefstra et al., 2012).

### 1.1 The Satisfaction of Cancer Patients

Cancer is a disease associated with death, because of its characteristics, i.e. typically poor prognosis and rapid evolution (Zacharakis, 2005). However, progress has been made in recent years in research and treatment of this disease (new chemotherapy and radio-diagnostic methods, specific tests for early diagnosis, etc.), results to treat certain types of cancer or significantly extending the life of the patient. In this context, the oncology patients after diagnosis of disease or subjecting them to surgery to remove the tumour, followed by systemic treatment by subjecting them to chemotherapy or radiation are under constant medical supervision. Therefore, these patients because of the frequent use of health services acquire special relationship with the doctor who monitors and the health system in general. The determination of the factors affecting the satisfaction of cancer patients is of great importance since they are a special group of patients with particular psycho-emotional reactions (Bredart et al., 2003). In particular, the severity of disease makes patients often exhibit feelings of denial, anger, anxiety, fear and uncertainty for the effectiveness of treatment and exhibit symptoms of depression (frustration, sadness and pessimism about their situation) (Adamakidou, 2009; Matis et al., 2010; Giese-Davis et al., 2011). Within the framework of physical and emotional support for cancer patients, who experience a life-threatening illness and their feelings of pain and death, it is necessary doctors, nurses and other healthcare professionals to cooperate with their patients in order to take sufficient information about the state of their health and gain confidence in their treatment (Levinson et al., 2010; Uitterhoeve et al., 2010; Rao et al., 2007).

### 1.2 Aim of Research

The purpose of this research is to investigate patient satisfaction with the quality of health services provided at the outpatient department of the Greek Anti-Cancer Hospital. Patients’ opinions will highlight problems within the organization and assess its level of functioning in both morning and afternoon clinics, providing medical, nursing and administrative services, as well as in hotel infrastructure. It is important to highlight oncology patients’ expectations and assessment of the healthcare services they receive.

## 2. Method of the Survey

For data collection a structured questionnaire with closed answers in the form of five-point Likert scale was used. The overall patients’ satisfaction results from the summation of the scores of individual questions (1 = strongly agree to 5 = strongly disagree). The questions used to create the total score are those relating to making an appointment, 2 questions on arrival at hospital, 5 questions on waiting for the examination and all questions relating to examination by the doctor (total 23 questions). The score can be adjusted from 23 to 115. Higher value indicates greater satisfaction. This scale is used by researchers because it is easily understood by respondents and ensures impartiality, since it allows the existence of negatively worded questions (Labiris & Niakas, 2005). The questionnaire has been used previously in several patient satisfaction surveys (Aletras et al., 2006; Aletras et al., 2009). It includes questions related to demographic and social data users, and overall service process in the outpatient department of the hospital. In particular, questions relating to:

a) Demographic and social characteristics of participants, (age, gender, education, health),
b) Oncology patient satisfaction questionnaire.

The questionnaire was approved by the Scientific Council and the Board of Anti-Cancer Hospital which granted permission to conduct the study (No. 34 Hospital Board decision). The completion of the questionnaires in outpatient morning clinics was completed personally by patients, under the supervision of the Head of Office Reception Hospital. The time to complete the questionnaire ranged from an average of 8-10 minutes.

### 2.1 The Sample

The sample randomly consisted of 100 patients (Response Rate: 76%) who visited the outpatient morning department and 89 patients (Response Rate: 67%).

### 2.2 Statistical Analysis

The means, standard deviations and median (interquartile range) were used to describe the overall assessment score of satisfaction of the participants. We checked for whether quantitative variables follow a normal distribution with the ‘Kolmogorov-Smirnov’ test. To compare the overall satisfaction score as a quantitative variable between three or more different groups we used the ‘ANOVA’ method and a non -parametric test ‘Kruskal-Wallis’, while comparing the overall satisfaction score between two groups we used the ‘t-test’ and non- parametric testing of ‘Mann-Whitney’. To evaluate the validity of the internal consistency of the questionnaire, we calculated the coefficient of Cronbach a). The Cronbach a coefficient was a = 0.72, which is an acceptable reliability. Linear regression analysis was used for finding the independent factors associated with the dimensions of satisfaction that generated dependency coefficients (b) and their standard errors (SE). For quantitative variables that are not normally distributed, linear regression analyses were performed with the use of degrees (ranks). Significance levels are bilaterally and statistical significance was set at 0.05. The statistical program used for analysing the data was SPSS 19.0.

## 3. Results

Table 1 shows that 41.0% of patients treated in morning clinics were aged 45-65 years. Also, the largest proportion (87.0%) was women. Regarding educational level, 39.4% of patients addressed in morning clinics were illiterate or primary school graduates, 34.3% high school graduates and 26.3% had a degree from a college or graduate school. Finally, most patients (45.0%) considered themselves to be in good health condition, 16.0% very good, 34.0% moderate and only 3.0% and 2.0% very poor and poor respectively.

**Table 1:** Demographics of morning and afternoon clinics

		Morning clinics		Afternoon clinics	

		N	%	N	%
Age	18-44	24	24,0	12	13,3
	45-65	41	41,0	41	45,6
	>65	35	35,0	37	41,1
Sex	Men	13	13,0	32	35,6
	Women	87	87,0	58	64,4
Education	Illiterate / Primary	39	39,4	24	26,7
	Middle School /High School	34	34,3	41	45,6
	university/Master	26	26,3	25	27,8
Health condition	very good	16	16,0	16	17,8
	good	45	45,0	58	64,4
	average	34	34,0	14	15,6
	bad	3	3,0	2	2,2
	very bad	2	2,0	0	0,0

Table 2 shows comparison of satisfaction scores between the morning and afternoon outpatient department. In particular, Table 2 shows the satisfaction scores of the participants according to their outpatient clinic visited.

**Table 2:** Comparison of morning and afternoon clinics

	Morning clinics	Afternoon clinics	P Mann-Whitney

	MEAN	MEAN	
Satisfaction score on the closing date	14,3±1,6	17,6±1,4	<0,001
Satisfaction score on arrival at the outpatient department	13,5±1,2	15,3± 1,3	<0,001
Satisfaction score on waiting for the examination	17,7±0,9	19,6±1,1	<0,001
Satisfaction score on the surgery and medical examination	47,5 ±3,6	53,3±3,9	<0,001
Overall satisfaction score, mean ± SD	85,6±7,6	97,5±4,7	<0,001*

* Student’s t-test

In particular, Table 2 shows that patients who received treatment at the afternoon clinic had significantly higher satisfaction score in scheduling an appointment and by the arrival in outpatient department compared to patients who visited the outpatient morning clinics. These patients are more satisfied because the appointment at the afternoon clinic are closely controlled by the patient himself (94.4% had to wait less than a month after making an appointment to the test), unlike the morning clinics where outpatient waiting time is usually higher (patient satisfaction stands at 58.0%). Moreover, the workload of the morning outpatient results in 16.0% and 40.0% of patients who complained about the process of making appointment and the choice of day and time of the appointment, compared with the afternoon clinics where the figures stood at around 0.0% and 15.5% (Table 3).

**Table 3:** Reasons of dissatisfaction between morning and afternoon clinics

	Morning clinics %	Afternoon clinics %
Less than a month waiting time until the appointment	58.0	94.4
Complained about the process of making appointment	16.0	0.0
Complained about the choice of day and time of the appointment	40.0	15.5

The rate of patient satisfaction by the staff of the Secretariat of outpatient department was really high. Regarding the arrival at the hospital in the afternoon clinics patients reach the hospital more often by car (48.9%) and are finding it easier to access it (91.1%) and to park their car (29.6%) compared with patients that are visiting morning clinics (the corresponding figures are around 30.0% to 56.6% and 20.7%).

Visitors to the afternoon clinics had significantly higher satisfaction scores on waiting for examination, compared with patients who visited the morning clinics. These patients reported more satisfaction because the waiting time from the scheduled appointment until contact with the doctor for examination usually ranged from 5 to 14 minutes (37.8%), while the usual waiting time in morning clinic ranges from 15 to 29 minutes (30.0%). Moreover, a significant percentage of patients waited in morning clinics from 30-59 minutes or more than one hour (22.0% and 18.0% respectively), where the corresponding percentages in the afternoon clinics are 15.6% and 4.4%. Therefore, 45.5% of patients who visited the clinic in the morning reported they waited too long in contrast to afternoon surgeries patients who reported they waited too long only 14.4% of the time. Regarding the comfort and cleanliness of the waiting area, afternoon patients reported more satisfaction. The cleanliness satisfaction scores were significantly higher for patients who come to the afternoon clinics compared with patients who attended the morning clinics. Satisfaction was based on the medical examination and the behaviours of the medical staff. Specifically, 28.0% of the visitors of the morning clinics felt the testing area was too narrow and uncomfortable, while only 2.2% of patients who visited the clinics in the afternoon expressed these complaints. Moreover, one of the main reasons for the higher patient satisfaction score of the afternoon clinics is the longer duration of time the physician’s spent with them. The examinations usually ranging from 11 to 20 minutes (34.4%) or 21-30 minutes (33.3%). Instead, the usual examination time in morning clinics is 6-10 minutes (48.0%) or 11-20 minutes (36.0%). Therefore, visitors to the afternoon clinics report being satisfied by the amount of time their physician spent with them (100.0%), while in the case of morning clinics, patient satisfaction rates was at 86.0% (percentage that is certainly high enough). Regarding the trust placed by patients in the outpatient hospital service, care by physicians and the skills and scientific relevance the study showed higher percentages of satisfaction in the afternoon clinics. However, the difference in satisfaction between morning and afternoon clinics was not significantly different. The figures of the morning clinic (e.g. 100.0% of visitors afternoon clinics were completely satisfied with the diagnosis, treatment and scientific competence of physicians, while the corresponding figures in morning clinics range in 97.0% and 89.0% respectively). The observed high levels of satisfaction in morning clinics was due to the kindness and helpfulness of the nursing staff (89.0% and 78.0% respectively), where the corresponding rates of satisfaction in afternoon clinics are 67.8% and 66.7%. Because of the workload of the laboratories in the morning hours, 31.5% of patients reported that they waited a significant amount of time in the waiting room until the examination, while those who attended the afternoon clinics the reported long waiting periods at 0.0%. It should be noted that only a small number of patients have laboratory testing ordered in afternoon, and when they do these labs are less time consuming procedures.

### 3.1 Overall Assessment Health Service

Patients were asked about their satisfaction with their visit overall in morning outpatient department. Table 4 shows the vast majority of patients (87.0%) were satisfied /satisfied from the services of the morning outpatient, while only 5.0% expressed the opposite view. The data also declare that all patients (100.0%) were completely satisfied / satisfied with the services of the afternoon clinics.

**Table 4:** Satisfaction with the overall visit in morning and afternoon outpatient department clinics

	Strongly agree	Agree	Neither agree nor disagree	Disagree	Strongly disagree

	N (%)	N (%)	N (%)	N (%)	N (%)
dissatisfied by visiting outpatient hospitals’ morning clinics (N= 100)	2 (2,0)	3 (3,0)	8 (8,0)	64 (64,0)	23 (23,0)
dissatisfied by visiting outpatient hospital’ afternoon clinics (N = 89)	0 (0)	0 (0)	0 (0)	23 (25,8)	66 (74,2)

The score of people who visited the outpatient mornings had average value of 85.6 (SD = 7.6). The minimum value was 58 and the maximum 103. The score them visited the Afternoon outpatient had average value of 97.5 (SD = 4.7). The minimum value was 84 and the maximum 105.

Table 5 describes the rates of overall satisfaction score of healthcare service depending on demographic and other factors.

**Table 5:** Rate overall satisfaction according to demographic and other factors in a morning and afternoon clinics

		Morning clinic Rate overall satisfaction			Afternoon clinic Rate overall satisfaction		

		mean	SD	p-value anova	mean	SD	p-value anova
**Age**	18-44	82,2	9,2	0,198	97,6	2,4	0,117
	45-65	86,0	7,5		96,0	5,0	
	>65	86,8	6,7		99,0	4,5	
**Sex**	Men	84,2	7,5	0,504*	99,1	4,2	0,070*
	Woman	85,8	7,7		96,6	4,8	
**Education**	Illiterate / Primary	86,9	6,5	0,577	97,7	5,0	0,215
	Middle School/High School	85,5	6,2		96,3	3,9	
	ΙΕΚ/ΤΕΙ-AEI/Master	84,8	8,3		99,0	5,1	
**Health condition**	Very good	84,9	7,9	0,758	96,3	2,4	0,693
	Good	86,1	8,9		97,7	4,5	
	Average	86,1	6,2		98,1	7,1	
	Bad	81,3	10,3				
	Very worst	81,0	5,7				

* t- test. Factors were not found to correlate significantly with overall satisfaction with the health services nor the mornings or in the afternoon outpatient.

## 4. Conclusions-Discussion

Cancer patient’s needs vary with time and according to the phase of the disease. Long waiting times are a major source of patient dissatisfaction and adversely affect patient compliance with treatment regimes and clinical outcomes. Cancer patients require longer consultation times, which have a build up effect of increasing waiting times of the other patients needing to be seen (Yeboah & Thomas, 2009). The satisfaction of cancer patients who utilize the services provided in the outpatient clinics of the Greek General hospital is high. Guests are more satisfied with the afternoon clinics. This data is in agreement with similar results (Labiris & Niakas, 2005; Matis et al., 2009; Gnardellis & Niakas, 2005; Giamalidis, 2009) who showed that the operation of these clinics has contributed to effectively improving the quality of services provided by hospitals and results in high satisfaction rates by patients. The overall satisfaction rate for the services provided in afternoon surgeries ranges between 97.5 to 85.6. The overall satisfaction score for the morning clinics, rates very high in view of the recent commencement of their operation.

It is worth noting that the highest rates of patient satisfaction, both in the mornings and in the afternoon clinics, is related to services provided by physicians (diagnosis, treatment, and instructions), their behaviour and their scientific competence. Given that oncology patients visiting the outpatient hospital are monitored at regular intervals by a particular doctor who knows their history, it is natural for patients to develop a special relationship of trust. This trust is reflected in the high levels of satisfaction reported.

These results are similarly observed by other researchers, that reported that these patients not only appreciate the scientific knowledge and skills of the physician, but also human contact with him, completeness of information and the time devoted to it, which are essential for the psychological and emotional support (Kyriacopoulos, 2009; Chandrinou et al., 2013). It should be noted that the patients of the afternoon clinics are more satisfied with the duration and conditions of examination, while a source of discontent for the patients of the morning surgery is the cramped space and lack of privacy and proper isolation during examination. Given that the mornings and afternoon clinics operate in the same area, the difference in the perception of the patients about the facilities and physicians’ discretion may be attributed to the greater movement of patients in morning clinics, and the number physicians and nurses working that creates the sense of crowding and tight space.

The large number of patients visiting the morning outpatient hospital compared to afternoon clinics, directly results in longer waiting times. Another contributing factors is not making appointments until the day of examination and lack of choices about the day and time of appointment and the long waiting time the patient sits in the waiting room until their turn for examination (Vastardis, 2009), These problems in the operation of the outpatient morning clinic, are a source of dissatisfaction for patients who seek the higher quality services provided in the afternoon clinics.

These confirm the results found in other Greek literature on patient satisfaction and demonstrate that one of the major problems in the organization and operation of outpatient morning clinic is the waiting list and the long waiting time for patients on the day of visit. These factors directly affect their satisfaction with the services provided.

No statistically significant relationship was found between overall satisfaction with the demographic characteristics of patients or other parameters. However, the analysis of individual parameters that constitute the overall service process of the outpatient hospital showed that the rating of services depends on patients’ state of health. Specifically, individuals who considered themselves to have very good state of health rated significantly lower process for making an appointment in morning outpatient versus subjects that characterized the state of their health good, fair, poor or very poor. The highest score and therefore more satisfaction were reported by those that reported a good state of health.

Also overall service process of the outpatient hospital showed that the rating of services depends on the educational level patients who have graduated with Vocational/Technical/Higher and hold postgraduate degrees reported higher scores and thus greater satisfaction from the process of making an appointment in the afternoon clinic as compared with illiterate and primary school graduates who had significantly lower scores and felt less satisfied.

Overall service process of the outpatient hospital also showed that the rating of services depends on the sex of patients, with females having significantly lower scores and thus less satisfaction from the surgery and medical examination in afternoon surgeries than male.

It is clear from what was explained in detail above, the measurement of patient satisfaction is a key methodological tool used to identify problems and improve the quality of services provided by health care providers. The present study showed high satisfaction rates for oncology patients visiting the mornings and afternoon clinics but highlighted some problems which should focus on the management of the hospital in order to further improve the quality of service. Given that the patients’ expectations and their interest in quality services are constantly increasing, the future of health services providers will belong to those suppliers who can adapt their services to meet patients’ needs. Therefore, a primary priority for public health agencies should be the adoption of appropriate management methods for the assessment, safeguarding and promoting the quality of service in order to be competitive with the private health sector that has experienced rapid growth in the country for the past few years. Those improvements have been mainly because of better organization, rapid service and the best hotel infrastructure. Separating outpatients into cancer clinics and non-cancer clinics may be a way of reducing clock waiting times in outpatient clinics and thus improving the quality of care to cancer patients.

**Limitation of the Study**

Relatively small sample size may have contributed to limited correlations. It is argued that the dissatisfaction, to the level that it has been documented, perhaps is due to other structural problems of the Greek health system.
